# Lattice layout and optimizer effect analysis for generating optimal transcranial electrical stimulation (tES) montages through the metaheuristic L1L1 method

**DOI:** 10.3389/fnhum.2024.1201574

**Published:** 2024-02-29

**Authors:** Fernando Galaz Prieto, Maryam Samavaki, Sampsa Pursiainen

**Affiliations:** Computing Sciences, Faculty of Information Technology, Tampere University, Tampere, Finland

**Keywords:** transcranial electrical stimulation (tES), optimization, linear programming, L1-norm, Interior-Point, metaheuristics

## Abstract

**Introduction:**

This study focuses on broadening the applicability of the metaheuristic L1-norm fitted and penalized (L1L1) optimization method in finding a current pattern for multichannel transcranial electrical stimulation (tES). The metaheuristic L1L1 optimization framework defines the tES montage via linear programming by maximizing or minimizing an objective function with respect to a pair of hyperparameters.

**Methods:**

In this study, we explore the computational performance and reliability of different optimization packages, algorithms, and search methods in combination with the L1L1 method. The solvers from Matlab R2020b, MOSEK 9.0, Gurobi Optimizer, CVX's SeDuMi 1.3.5, and SDPT3 4.0 were employed to produce feasible results through different linear programming techniques, including Interior-Point (IP), Primal-Simplex (PS), and Dual-Simplex (DS) methods. To solve the metaheuristic optimization task of L1L1, we implement an exhaustive and recursive search along with a well-known heuristic direct search as a reference algorithm.

**Results:**

Based on our results, and the given optimization task, Gurobi's IP was, overall, the preferable choice among Interior-Point while MOSEK's PS and DS packages were in the case of Simplex methods. These methods provided substantial computational time efficiency for solving the L1L1 method regardless of the applied search method.

**Discussion:**

While the best-performing solvers show that the L1L1 method is suitable for maximizing either focality and intensity, a few of these solvers could not find a bipolar configuration. Part of the discrepancies between these methods can be explained by a different sensitivity with respect to parameter variation or the resolution of the lattice provided.

## 1 Introduction

Transcranial Electrical Stimulation (tES) is a non-invasive brain stimulation method used for stimulating neuronal activity, treating psychiatric disorders, and studying neuronal behavior by transmitting a constant low-intensity current pattern through a set of electrode patches attached to the scalp of the subject to modulate cortical excitability (Nitsche and Paulus, 2000). In tES, a volumetric current density in the brain is generated by injecting through the scalp a current pattern that can be described via different properties, including the number of active electrodes, their physical description (e.g., positioning, shape, permittivity, and impedance values), the applied stimulus waveform (e.g., amplitude, pulse shape, pulse width, and polarity), the number of stimulation sessions, and the time interval (Peterchev et al., 2012). Since different electrode montages result in distinct brain current flow, clinicians and researchers can adjust the montage to target or avoid specific brain regions in an application-specific manner.

An increasingly popular form of tES is the Transcranial Direct Current Stimulation (tDCS) method (Paulus, 2011; Moreno-Duarte et al., 2014; Thair et al., 2017; Reed and Cohen Kadosh, 2018). Compared to other non-invasive stimulation methods, the advantages of tDCS can be attributed to its inexpensive and approachable characteristics. Unlike the intricate machinery required for Transcranial Magnetic Stimulation (TMS) or the specialized frequency considerations in Transcranial Alternating Current Stimulation (tACS), tDCS involves a simpler setup—a direct current passed through scalp electrodes. This simplicity not only reduces the cost of equipment but also enhances portability, making tDCS more accessible for various settings, including home use. The simplicity and minimal training required contribute to its user-friendly nature enabling a broader range of individuals to utilize or participate in studies involving this method. Whereas tDCS is classically applied in a two-channel configuration (Kaufmann et al., 2021), its focality can be enhanced via multiple channels, which has motivated the introduction of advanced optimization methods for finding an optimal multi-channel montage (Fernandez-Corazza et al., 2020).

tES modeling involves constructing computational representations of the head and brain anatomy, simulating the distribution of electric fields. This process integrates factors such as electrode placement, tissue conductivity, and finite element method simulations to visualize and analyze the spatial distribution of the electric field within the brain. Generating a high-resolution forward model is critical for building an explicit patient-specific head model, determining optimal positioning of electrodes, and predicting electric field generation across the brain for specific stimulation configurations (Faria et al., 2011; Rampersad et al., 2013; Wagner et al., 2013). Using such a forward model, multi-electrode stimulation can be optimized via specifically designed mathematical methodology (Dmochowski et al., 2011; Ruffini et al., 2014; Guler et al., 2016; Wagner et al., 2016; Fernandez-Corazza et al., 2020), such as the recently developed convex optimization schemes including the Distributed Constrained Maximum Intensity (D-CMI) (Khan et al., 2022), and the metaheuristic L1-norm regularized L1-norm fitting (L1L1) (Galaz Prieto et al., 2022) which aim at an individualized distributional fit for a given target activity.

In this study, we aim to broaden the applicability of the linear programming (LP)-based L1L1 method for finding tES electrode montages computationally in a comprehensive manner, i.e., by evaluating the metaheuristic results and total computing time through different mathematical optimization algorithms and packages; this includes Interior-Point (IP) (Mehrotra, 1992), Primal-Simplex (PS), and Dual-Simplex (DS) (Boyd and Vandenberghe, 2004) as alternative LP algorithms, and Matlab (R2020b) from MathWorks (Zhang, 1999), MOSEK Optimization Suite (Release 9.0) (Mosek, 2019), Gurobi Optimization (9.5.1) (Gurobi Optimization LLC, 2022), SDPT3 (4.0) (Tütüncü et al., 2003), and SeDuMi (1.3.5) (Sturm, 1999; Frenk et al., 2000; Polik et al., 2007) as alternative packages. The latter two open-source alternatives are available in the CVX optimization toolbox (Grant and Boyd, 2014). We also investigate the metaheuristic hyperparameter optimization (HPO) task of L1L1 via exhaustive search (Bianchi et al., 2009) and recursive search (Je and Park, 2013) with heuristic direct search as a reference algorithm (Bogani et al., 2009).

Our results suggest that the performance differences between the above-mentioned optimization packages, algorithms, and search methodology can be crucial regarding the optimization results, focality stimulation current, and the availability of active channels in the montage. Moreover, exhaustive and recursive search methods can also be considered preferable to heuristic direct search in terms of their overall reliability and predictability.

## 2 Materials and methods

In tES, a real *L* × 1 current pattern **y** is injected into the subject's head through a set of contact electrodes attached to the scalp. These electrodes, ranging from 0.5 to 4.0 milliamperes (mA) (Zaghi et al., 2010; Khadka et al., 2020; Workman et al., 2020), form what is known as an electrode montage and are responsible for distributing the injected volumetric current density–measured in ampere per square meter (A/m^2^)–throughout the scalp, skull, cerebrospinal fluid (CSF), and brain components, including cortical and subcortical brain structures. The governing linear system is of the form


(1) $$\begin{array}{l} \hat{\text{L}}\text{y}=\hat{\text{x}}, \end{array}$$

where $\hat{\text{L}}$ is a real *N* × *L* lead field matrix (forward mapping) that describes the relationship between the **y**, and $\hat{\text{x}}$ is a real *N* × 1 discretized volume current density vector. The linear system (Equation 1) is re-interpreted component-wise as the focused field ${\hat{\text{L}}}_{1}\text{y}={\hat{\text{x}}}_{1}$, where the target field has non-zero values, and the nuisance field ${\hat{\text{L}}}_{2}\text{y}=\text{0}$, where it vanishes. Detailed mathematical definition of the lead field matrix refer to *Appendix A. Forward model* in Galaz Prieto et al. (2022).

The optimization problem needs to find the best matching between **y**, and the focused field via **Ly** = **x**, where the projection of the focused field into the direction of the target constitutes the first component as


$$\begin{array}{lll} \text{L} & =\left(\begin{array}{c} {\text{L}}_{1}\\ {\text{L}}_{2} \end{array}\right)=\left(\begin{array}{c} \text{P}{\hat{\text{L}}}_{1}\\ {\hat{\text{L}}}_{2} \end{array}\right)\;\text{and}\; & \text{x}=\left(\begin{array}{c} {\text{x}}_{1}\\ \text{0} \end{array}\right)=\left(\begin{array}{c} \text{P}{\hat{\text{x}}}_{1}\\ \text{0} \end{array}\right) \end{array}$$

with **P** denoting a matrix that projects a vector into the direction of ${\hat{\text{x}}}_{1}$. The target amplitude ||**x**_1_||_2_ is set as 3.85 A/m^2^ which is an approximation of the excitation current threshold for nerve fibers of the upper limb area of the motor cortex (Kowalski et al., 2002).

### 2.1 L1-norm fitted and regularized optimization

The goal in L1-norm Fitted and Regularized (L1L1) optimization method (Galaz Prieto et al., 2022) is to minimize


(2) $$\begin{array}{l} \underset{y}{\min}\{\|\left(\begin{array}{c} L_{1}y-x_{1}\\ \Psi_{\varepsilon }[\nu^{-1}L_{2}y] \end{array}\right){\|}_{1}+\alpha \zeta \|y{\|}_{1}\},\\ \text{s}.\text{t}.\;y\gamma 1,\;\|y{\|}_{1}\leq \mu ,\;\sum_{\ell =1}^{L}y_{\ell }=0. \end{array}$$

The injection on every active ℓ-th electrode channel is limited to γ ≤ 2.0 mA, the total injection current dose flowing through the tES head cap is within the safety limit μ ≤ 4.0 mA, and the total sum of electric current from every active electrode channel in *y*_ℓ_, where ℓ ∈ {1, ⋯ , *L*}, must be equal to zero. The regularization parameter **α** sets the level of L1-regularization with respect to the scaling value ζ = ||**L**||_1_. The function


$$\begin{array}{l} {\text{Ψ}}_{\varepsilon }{\left[\text{w}\right]}_{m}=\max\left\{|w_{m}|,\varepsilon \right\}\text{ for }m=\left\{1,2,\cdots \;,M\right\}, \end{array}$$

where **w** = (*w*_1_, *w*_2_, ⋯ , *w*_*M*_), sets the nuisance field threshold 0 ≤ ε ≤ 1 with respect to the scaling value ν = ||**x**||_∞_, meaning that entries (_**L**_2_**y**)*m*_ with an absolute value below εν do not actively contribute to the minimization process due to the threshold. We refer to the set {*m*:|(_**L**_2_**y**)*m*_| ≥ εν} as the *constraint support*, i.e., the index set contributing to the value of the objective function. Detailed formulation of the linear programming system (Equation 2) can be found in Galaz Prieto et al. (2022).

The current density Γ of the focused field is defined as


$$\begin{array}{l} \Gamma =\frac{{\text{x}}_{1}^{T}{\text{L}}_{1}\text{y}}{||{\text{x}}_{1}{||}_{2}}\text{ and }\Gamma_{\text{max}}=\arg\underset{\text{y},\alpha ,\varepsilon }{\max}\Gamma \;, \end{array}$$

and the focality of the stimulus Θ is defined as the following current ratio


$$\begin{array}{l} \Theta =\frac{\Gamma }{||{\text{L}}_{2}\text{y}{||}_{2}/\sqrt{M}}\;and\;\Theta_{max}=\arg\underset{\text{y},\alpha ,\varepsilon }{\max}\Theta . \end{array}$$

The metacriterion Γ ≥ Γ_0_ is applied to maintain appropriate intensity at the target location. Namely, without a lower bound for the intensity, the intensity of the maximizer is likely to vanish.

### 2.2 Two-stage metaheuristic lattice search

**Figure 1:**
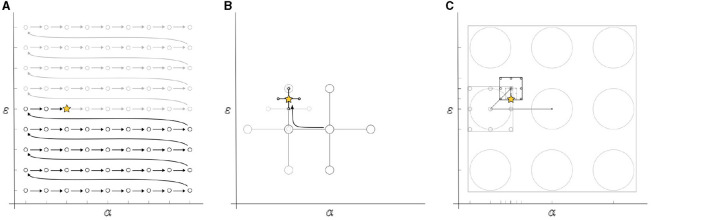
Schematic illustration of the Hyperparameter Optimization (HPO) techniques applied; **(A)** the exhaustive search method evaluates the entire hyperparameter space, case by case; **(B)** The direct search method employs a pattern that shrinks in size towards the direction in which the objective function decays; and **(C)** the recursive search divides the space into subspaces based on the size of the search lattice, shrinking and repositioning the lattice towards the most fitting solution in a recursive manner.

To derive a multi-channel tES montage following the aforementioned equations, the optimization framework takes into account the following indications: (A) a procedure for selecting the most relevant electrodes in the montage for a given region of interest; (B) a definition of the tuning parameters which will maximize or minimize the objective function; and (C) a method to evaluate said parameters and retrieve data (search method). In this study, 128 electrodes were attached to the scalp following the international 10-10 EEG hardware system with an impedance of 2.0 kOhm (kiloohms). Physiological impediments in the head model, fluctuation in conductivity tissue, and behavior of the injected current aspects are excluded. The framework of this search is as follows:

(A) The *two-stage* determines which of the tES channels in the neurostimulator headgear should be set as active or inactive based on the field distribution on the head surface for a given current source in the brain. After calculating the lead field matrix, the user specifies an approximate region of interest through forward dipole modeling (Bauer et al., 2015; Medani et al., 2015; Pursiainen et al., 2016). This is the highlighted region from which the two-stage procedure shall prioritize the electrode selection as follows:
  (A.1). During the first stage, the optimization model sets all channels with an initial current of zero value and determines a volumetric current density influenced by the electric properties, direction, and positioning of the dipole modeling. Then, the optimization model filters the montage down to a (user-defined) number of electrodes that contribute the most to the maximal safety tES current injection based on the initial range of α and ε values provided. The corresponding electric potential from the now-limited montage with channels *y*_ℓ_ is normalized to meet the intended maximum current injection μ value while the remaining electrodes are opted out of further calculations. We constraint the total number of active electrodes available to ℓ = 20 inspired by commercial tES systems (Roy et al., 2019; Tost et al., 2021).
  (A.2). In the second stage, the optimization re-runs using only the active electrodes obtained previously. In this stage, the objective function can be retroactively modified to retrieve a customized montage that favors an intense volumetric current density Γ or a maximal stimulation focality given a target current Θ. The final result is then thresholded to a non-zero number of currents in the pattern.
(B) Using *metaheuristic* methodology means developing an algorithm that can produce near-optimal results in a computationally feasible time (Bianchi et al., 2009). In the present context, the objective is to iteratively adjust the parameters α and ε to ascertain a solution that minimally impairs the objective function. The aim is to secure a heightened amplitude within the targeted focus field while concurrently mitigating undesirable signals (the nuisance field). We define a parameter space by specifying ranges for α_*m*_ from -100 to -20 dB and ε_*n*_ from -160 to 0 dB, employing logarithmic increments. Plotting these parameter values on a Cartesian plane elucidates the search space κ, subject to a set of constraints delineated by the linear programming paradigm at hand.
(C) The *lattice search* aspect defines the instructions on how to retrieve information from the search space *κ* for solving (Equation 2). This task can be considered as a hyperparameter optimization (HPO) exercise (Feurer and Hutter, 2019; Yang and Shami, 2020) for building a predictive model that performs best when using the most fitting α_*m*_ and ε_*n*_ parameters. The following exploration techniques are evaluated for finding these parameters: *exhaustive search, direct search*, and *recursive search*.
  (C.1) The *exhaustive search*, or grid search, systematically evaluates every possible candidate solution within the search space κ, i.e., the Cartesian product of each α_*m*_ and ε_*n*_ value in existence (Figure 1A). The final candidate solution is the combination that best minimizes the objective function. We applied a coarse grid of size *κ* = 15 and compared it against a finer grid of size *κ* = 40.
  (C.2) By *direct search*, we refer to the Generalized direct search (GPS) (Bogani et al., 2009) available in the Matlab's optimization toolbox. It aims at finding a point in the hyperparameter space without knowledge of any gradient. The method begins with a given search window *D*^(*i*)^ and an initial estimate $\psi_{\left(\alpha ,\varepsilon \right)}^{\left(i\right)}$ acting as a pivot. The location of this window is centralized over the pivot along with its four orthogonal neighbor points in the Euclidian distance *w*^(*i*)^, i.e.,
$$\begin{array}{r} D^{\left(i\right)}=\{\psi_{\left(\alpha_{m},\varepsilon_{n}\right)}^{\left(i\right)},\psi_{\left(\alpha_{m},\varepsilon_{n+w^{\left(i\right)}}\right)}^{\left(i\right)},\\ \psi_{\left(\alpha_{m+w^{\left(i\right)}},\varepsilon_{n}\right)}^{\left(i\right)},\psi_{\left(\alpha_{m},\varepsilon_{n-w^{\left(i\right)}}\right)}^{\left(i\right)},\psi_{\left(\alpha_{m-w^{\left(i\right)}},\varepsilon_{n}\right)}^{\left(i\right)}\}. \end{array}$$
    Figure 1B depicts the mesh and its behavior. At each *i*-th iteration within the mesh, if a neighboring point performs better than the center point, the window reallocates this point as the new pivot. If none of these points yields a better output, then the length of the mesh *w* is reduced, and a new set of neighbor points is adopted. That is,
$$w^{\left(i+1\right)}=\{\begin{array}{ll} w^{\left(i\right)}, & \text{if }\psi_{\left(\alpha_{m},\varepsilon_{n}\right)}^{\left(i+1\right)}\leq \psi_{\left(\alpha_{m},\varepsilon_{n}\right)}^{\left(i\right)},\\ w^{\left(i\right)}/2, & \text{if none satisfies}. \end{array}$$
    The cycle repeats until the number of *i* iterations is reached or the algorithm is unable to find any better point.
  (C.3) The *recursive search* is a modified version of the three-step search block-matching algorithm (Je and Park, 2013) that resembles a combination of the previously mentioned methods; it defines the subset of the hyperparameter space as in (C.1), and converges towards the most fitting solution by recursively reducing the region of feasibility similar to (C.2). In this study, we adapted the algorithm for tuning α and ε by dividing these finite sets into two linearly-spaced vectors with $\left\{\tilde{\kappa }\right\}$ points, recursively through a number of *M* iterations, taking their minimum and maximal values as their lower and upper bounds, i.e.,
$$\begin{array}{ll} {\tilde{\alpha }}^{\left(M+1\right)} & =\left\{\left(\alpha_{\tilde{\kappa }}^{\left(M\right)}-\alpha_{1}^{\left(M\right)}\right)/\left(\tilde{\kappa }-1\right)\right\}\text{, and}\\ {\tilde{\varepsilon }}^{\left(M+1\right)} & =\left\{\left(\varepsilon_{\tilde{\kappa }}^{\left(M\right)}-\varepsilon_{1}^{\left(M\right)}\right)/\left(\tilde{\kappa }-1\right)\right\}\text{, respectively.} \end{array}$$
    Thus, the method updates the hyperparameter space by replacing it with a narrower subspace instead of shrinking the search window (Figure 1C). At each *M*-th iteration, the search window, with initial size $w_{i}=\beta_{i}{\;}^{\text{2}}$, finds the center of the subspace such that
$$\begin{array}{l} \frac{1}{\beta_{i}}\psi^{\left(i\right)}\leq \psi^{\left(i\right)}\leq \beta_{i}\psi^{\left(i\right)}, \end{array}$$
    where ψ^(*i*)^ is the central point at the *i*-th grid and the optimal solution from the previous (or initial) feasible region $\beta_{i-1}^{-1}\psi^{\left(i-1\right)}\leq \psi^{\left(i-1\right)}\leq \beta_{i-1}\psi^{\left(i-1\right)}$. A search window of size *w*_*i*+1_ is centered at the location of ψ^(*i*)^, i.e., β_*i*_ = *sβ*_*i*−1_ with *s*>0,
$$\begin{array}{l} w_{M}=\beta_{M}^{2}={\left(\frac{u_{0}}{l_{0}}\right)}^{1/K}\;and\;s={\left(\frac{l_{0}}{u_{0}}\right)}^{\frac{K-1}{KM}}, \end{array}$$
    where *u*_0_ and *l*_0_ are the upper and lower limits from the initial hyperparameter space, respectively, and *K* equals a user-defined reference lattice size for a single non-recursive search. We evaluate and compare this method by setting $\tilde{\kappa }=\left\{3,5,7,9\right\}$, with *M* = {1,  ⋯, 3} in each case. With this set of equations, the workload of an exhaustive search is reduced to $O\left(M{\tilde{K}}^{2}\right)$, where $\tilde{K}$ is a smaller grid size, i.e, $\tilde{K}<\frac{K}{\sqrt{M}}$.

Additionally, we estimated the limits for the lattice-induced deviation of Θ_max_ and Γ_max_ via a second-order Taylor's polynomial approximation (Sauer, 2018), With this strategy, the deviation is obtained with respect to a hypothetical lattice with twice the resolution compared to the actual one.

### 2.3 Reciprocity principle

The Reciprocity Principle (Fernandez-Corazza et al., 2020) is an explicit approach for obtaining maximum current density, Γ_max_, based on the reciprocity of the electromagnetic field propagation. Specifically, the maximum stimulation amplitude is obtained with a two-patch tES electrode montage corresponding to the two greatest EEG electrode voltages generated by a desired target current in the brain. The principle considers the connection between the forward and reverse propagation of the electromagnetic field, which is predicted by the lead field matrix.

#### 2.3.1 Formulation of the reciprocity principle for a tES lead field matrix

While gradient propagation in general electromagnetism is not always reciprocal, it can be shown that a bipolar montage in tES corresponds to the greatest absolute back-projected currents in the vector $L^{T}x_{1}$. The reciprocity principle can be formulated, for a restricted system, as


(3) $$\begin{array}{l} \text{L}{\text{R}}_{K}{\text{y}}_{K}=\text{x}, \end{array}$$

where **R**_*K*_ denotes a real *N* × *K* (*K* ≤ *N*) restriction matrix whose nonzero entries *r*_*i*_*j*_, *j*_ = 1 correspond to an ordered subset of electrodes


$$\begin{array}{r} S=i_{j}:j=1,2,\cdots \;,K,\text{with}|{\left({\text{L}}_{1}^{T}{\text{x}}_{1}\right)}_{i_{1}}|\geq |{\left({\text{L}}_{1}^{T}{\text{x}}_{1}\right)}_{i_{2}}|\geq \cdots \\ \geq |{\left({\text{L}}_{1}^{T}{\text{x}}_{1}\right)}_{i_{K}}|. \end{array}$$

The reciprocity principle follows by writing the intensity as $\Gamma =\sigma_{K}{\text{y}}_{K}^{T}{\text{s}}_{K}$ with $\sigma_{K}=||{\text{R}}_{K}{\text{L}}_{1}^{T}{\text{x}}_{1}{||}_{1}/||{\text{x}}_{1}{||}_{2}$ and ${\text{s}}_{K}={\text{R}}_{K}{\text{L}}_{1}^{T}{\text{x}}_{1}/||{\text{R}}_{K}{\text{L}}_{1}^{T}{\text{x}}_{1}{||}_{1}$ describing that Γ can be interpreted as a projection of **y**_*K*_ on σ_*K*_**s**_*K*_. Thus, the maximum of Γ is achieved when **y**_*K*_ is parallel to **s**_*K*_. The maximizer is then up-scaled to match the applied current dose μ, i.e., **y**_*K*_ = μ**s**_*K*_. Therefore, the corresponding maximum intensity is $\Gamma =\mu \sigma_{K}||{\text{s}}_{K}{||}_{2}^{2}$. The optimal maximizer montage is


$$\underset{K}{\max}\mu \sigma_{K}||{\text{s}}_{K}{||}_{2}^{2},$$

where, by definition, ||**s**_*K*_||_1_ = 1 for any *K* = 1, 2, …, *N*, and the entries of **s**_*K*_ are ordered in descending order with respect to their absolute value. Assuming that these entries are given by λ_1_≥λ_2_≥⋯≥λ_*K*_≥0, respectively, it holds that $||{\text{s}}_{K}{||}_{1}=\overset{K}{\underset{j=1}{\sum }}\lambda_{j}$, $||{\text{s}}_{K-1}{||}_{1}={\left(\lambda_{j}-1\right)}^{-1}\overset{K-1}{\underset{j=1}{\sum }}\lambda_{j}$, and


$$\begin{array}{ll} ||{\text{s}}_{K}{||}_{2}^{2}-||{\text{s}}_{K-1}{||}_{2}^{2} & =\frac{\lambda_{K}}{1-\lambda_{K}}\left(\lambda_{K}^{2}-\lambda_{K}+\frac{2-\lambda_{K}}{1-\lambda_{K}}\overset{K-1}{\underset{j=1}{\sum }}\lambda_{j}^{2}\right)\geq \\ \;\frac{\lambda_{K}}{1-\lambda_{K}}\left(\lambda_{K}^{2}-\lambda_{K}+2\overset{K-1}{\underset{j=1}{\sum }}\lambda_{j}^{2}\right)\\ \;\geq \frac{\lambda_{K}}{1-\lambda_{K}}\left(K\lambda_{K}^{2}-\lambda_{K}+\overset{K-1}{\underset{j=1}{\sum }}\lambda_{j}^{2}\right). \end{array}$$

The equality follows a straightforward substitution, the first inequality is based on


$$\frac{2-\lambda_{K}}{1-\lambda_{K}}=1+\frac{1}{1-\lambda_{K}}\geq 2,$$

and the second one is obtained as $\left(K-1\right)\lambda_{K}^{2}\leq \overset{K-1}{\underset{j=1}{\sum }}\lambda_{i_{j}}^{2}$. Following from the discriminant, together with the Arithmetic Mean–Quadratic Mean inequality


$$\frac{1}{K-1}\overset{K-1}{\underset{j=1}{\sum }}\lambda_{j}^{2}\geq {\left(\frac{1}{1-K}\overset{K-1}{\underset{j=1}{\sum }}\lambda_{j}\right)}^{2},$$

The second factor in Equation (3) does not have roots if


$$\begin{array}{l} K\overset{K-1}{\underset{j=1}{\sum }}\lambda_{j}^{2}\geq {\left(\overset{K-1}{\underset{j=1}{\sum }}\lambda_{j}\right)}^{2}\geq \frac{1}{4},\;i.e.,\;\overset{K-1}{\underset{j=1}{\sum }}\lambda_{j}\geq \frac{1}{2}. \end{array}$$

This assumption is valid since a montage with only two active channels cannot contain more than two halves of the total dose (otherwise, the sum of said currents will be less than zero). Hence, $||{\text{s}}_{K}{||}_{2}^{2}-||{\text{s}}_{K-1}{||}_{2}^{2}\geq 0$ for any montage, and the maximum of Γ is obtained with the bipolar pattern that corresponds to the first two entries *i*_1_ and *i*_2_ in the set S.

### 2.4 Mathematical optimization software

We solve the optimization task (Equation 2) using the Interior-Point (IP), the Primal-Simplex (PS), and the Dual-Simplex (DS) methods. The class of the IP methods is sub-divided into the primal-dual algorithms (predictor-corrector) (Fiacco and McCormick, 1964; Mehrotra, 1992) and the barrier methods, which determine the feasible set via a barrier function. While IP methods utilize Newton's method to operate in the interior of a feasible set (Boyd and Vandenberghe, 2004), simplex methods seek solutions by considering the feasible set as a convex polytope and moving along its edges. While this strategy uses less memory than the interior-point strategy, it has lower predictability for large-problem convergence.

The concepts of primal- and dual-simplex refer to the formulation of the linear programming problem; by presenting the entries of the current pattern **y** as differences of non-negative variables (*y*_*i*_ = *s*_*i*_−*p*_*i*_, *s*_*i*_, *p*_*i*_≥0) and the equality constraint via two inequalities (condition *a* = 0 is satisfied, *a* ≤ 0 and −*a* ≤ 0), the task can be brought back to the following standard primal formulation:


$$\underset{\text{z}}{\max}{\text{c}}^{T}\text{z}\text{ subject to }\text{A}\text{z}\leq \text{b},\;z\geq 0,$$

whose dual is given by


$$\underset{\hat{\text{z}}}{\min}{\text{b}}^{T}\hat{\text{z}}\text{ subject to }{\text{A}}^{T}\hat{\text{z}}\geq \text{c},\;\hat{\text{z}}\geq 0.$$

The IP algorithms applied in this study include Gurobi's parallel barrier method and the primal-dual routines from Matlab, MOSEK, SDPT3, and SeDuMi. The simplex methods include MOSEK's PS and DS, Gurobi's PS and DS, and Matlab's DS algorithm. Matlab's Optimization Toolbox has two IP solvers, of which we apply the interior-point legacy (IPL), whose origin is in the Linear-Programming Interior Point Solvers (LIPSOL) package (Zhang, 1999). All the solvers, their types, and their abbreviations used in this study are described in Table 1.

**Table 1:** Description of the Linear Programming (LP) solvers applied for solving the L1L1 optimization problem through the Interior-Point (IP), Primal-Simplex (PS), and Dual-Simplex (DS) algorithms.

**Solver**	**Interface**	**Method**	**Code**
Matlab 2020b	Optimization toolbox	Interior-Point (Primal-Dual)	Matlab IP
		Primal-Simplex	Matlab PS
		Dual-Simplex	Matlab DS
MOSEK 9.0	MOSEK toolbox	Interior-Point (Primal-Dual)	MOSEK IP
		Primal-Simplex	MOSEK PS
		Dual-Simplex	MOSEK DS
Gurobi Optimizer	Gurobi toolbox	Interior-Point (Barrier Method)	Gurobi IP
		Primal-Simplex	Gurobi PS
		Dual-Simplex	Gurobi DS
SDPT3 4.0	CVX 2.1	Interior-Point (Primal-Dual)	SDPT3 IP
SeDuMi 1.3.5	CVX 2.1	Interior-Point (Primal-Dual)	SeDuMi IP

All solvers were embedded with Matlab's version R2020b and called by the optimizer of the Zeffiro Interface (ZI) toolbox. Matlab, MOSEK, CVX's SDPT3, and CVX's SeDuMi apply primal-dual routines, and Gurobi uses the barrier method.

### 2.5 Numerical domain and computing platform

As the domain of the numerical simulations, we applied a realistic tetrahedral 1.0 mm FE mesh based on an open T1-weighted Magnetic Resonance Imaging (MRI) dataset^1^. Through FreeSurfer Software Suite^2^, we segmented the data to find the complex surface boundaries between different tissue compartments, including the skin, skull, cerebrospinal fluid (CSF), gray and white matter, and subcortical structures such as brain stem, thalamus, amygdala, and ventricles (Fischl, 2012). Their conductivity values, which influence the accuracy of the forward solution (Montes-Restrepo et al., 2014), were set according to (Dannhauer et al., 2011). We discretized the volumetric current density to solve the inverse problem using 563 spatial nodes evenly distributed in the gray and white matter compartments of the cerebrum and cerebellum with approximately 1.3 cm (centimeters) distance between two neighboring nodes, associating each node with three divergence-free Cartesian field components.

**Figure 2:**
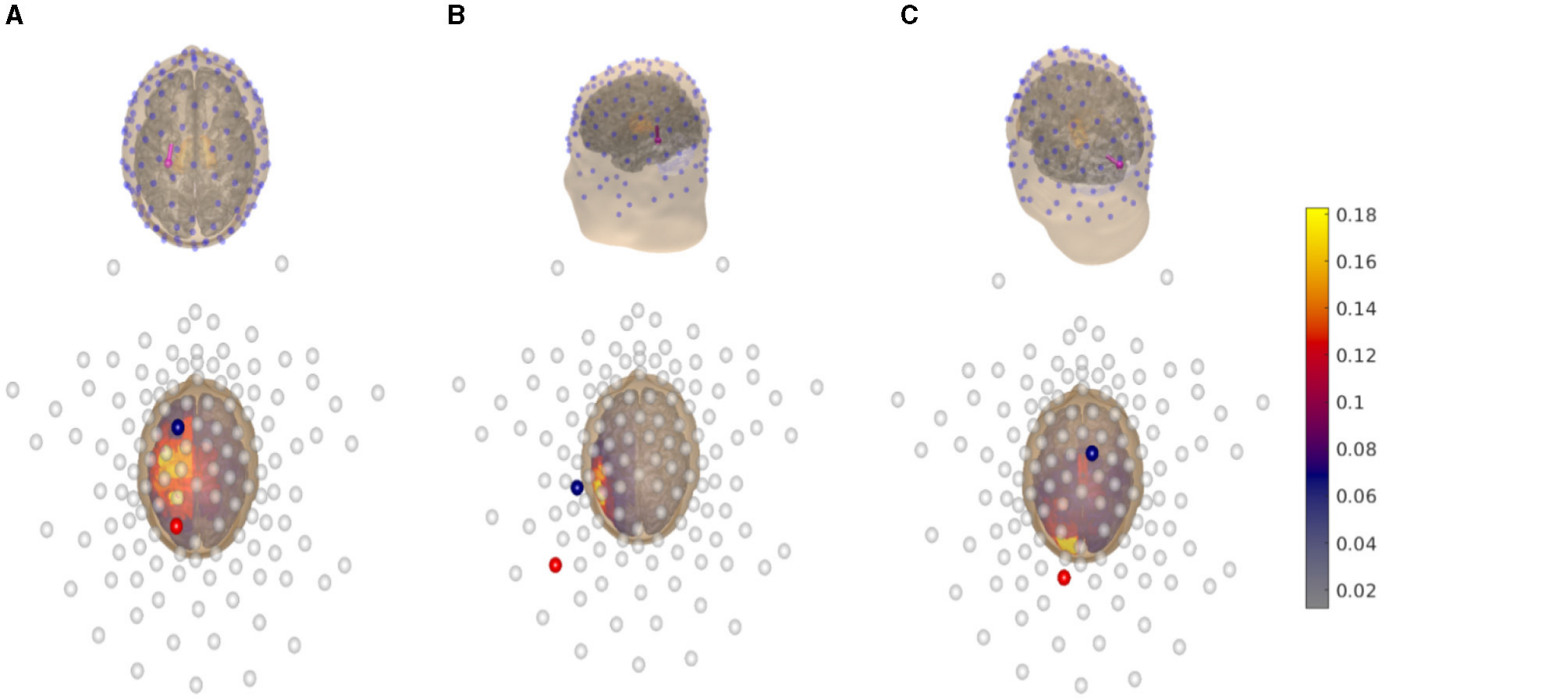
**Top row:** 3D view of the head model coupled with a 128-channel electrode array (blue dots) following a 10-10 EEG hardware configuration. A synthetic dipole, (magenta spherical arrow), which simulates a current distribution, is placed at the **(A)** primary somatosensory cortex in the postcentral gyrus, **(B)** primary auditory cortex in posterior superior temporal gyrus, and **(C)** primary visual cortex in the occipital lobe, respectively. **Bottom row:** 2D plane view of the head model displaying the electric field distribution generated by an optimal tES montage following the reciprocity principle which maximizes the focused volumetric current density. The direction of the injection current pattern generated by the montage matches the dipole's orientation. The anodal channels (red spheres) are found by the posterior, while the cathodal channels (blue spheres) by the parietal or frontal sections. The empty circles indicate inactive channels. The volumetric current density is given in Amperes per square meter (A/m^2^).

Through dipole modeling (Bauer et al., 2015; Medani et al., 2015; Pursiainen et al., 2016), we define the region of interests from which the multi-channel tES montage should be derived. We selected the primary somatosensory cortex in the postcentral gyrus (Figure 2A), the primary auditory cortex of the posterior superior temporal gyrus (Figure 2B), and the primary visual cortex in the occipital lobe (Figure 2C) as the target areas. Each dipole is normally oriented with respect to the surface of the gray matter to satisfy the normal constraint of brain activity in the cerebral cortex (Creutzfeldt et al., 1962). Each L1L1 method-based current pattern obtained represents an approximative solution to the optimization problem (Equation 2) corresponding to one of the aforementioned areas.

We performed the numerical simulations using a Dell 5820 workstation with a 10-core Intel Core i9-10900X processor and 256 GB of RAM. The L1L1 solver was implemented in Matlab-based Zeffiro Interface toolbox^3^ (He et al., 2019) which builds a high-resolution finite element (FE) mesh and generates a tES lead field matrix (Galaz Prieto et al., 2022) for a given surface-based head segmentation incorporating the Complete Electrode Model's (CEM) boundary conditions (Pursiainen et al., 2012, 2017).

## 3 Results

**Figure 3:**
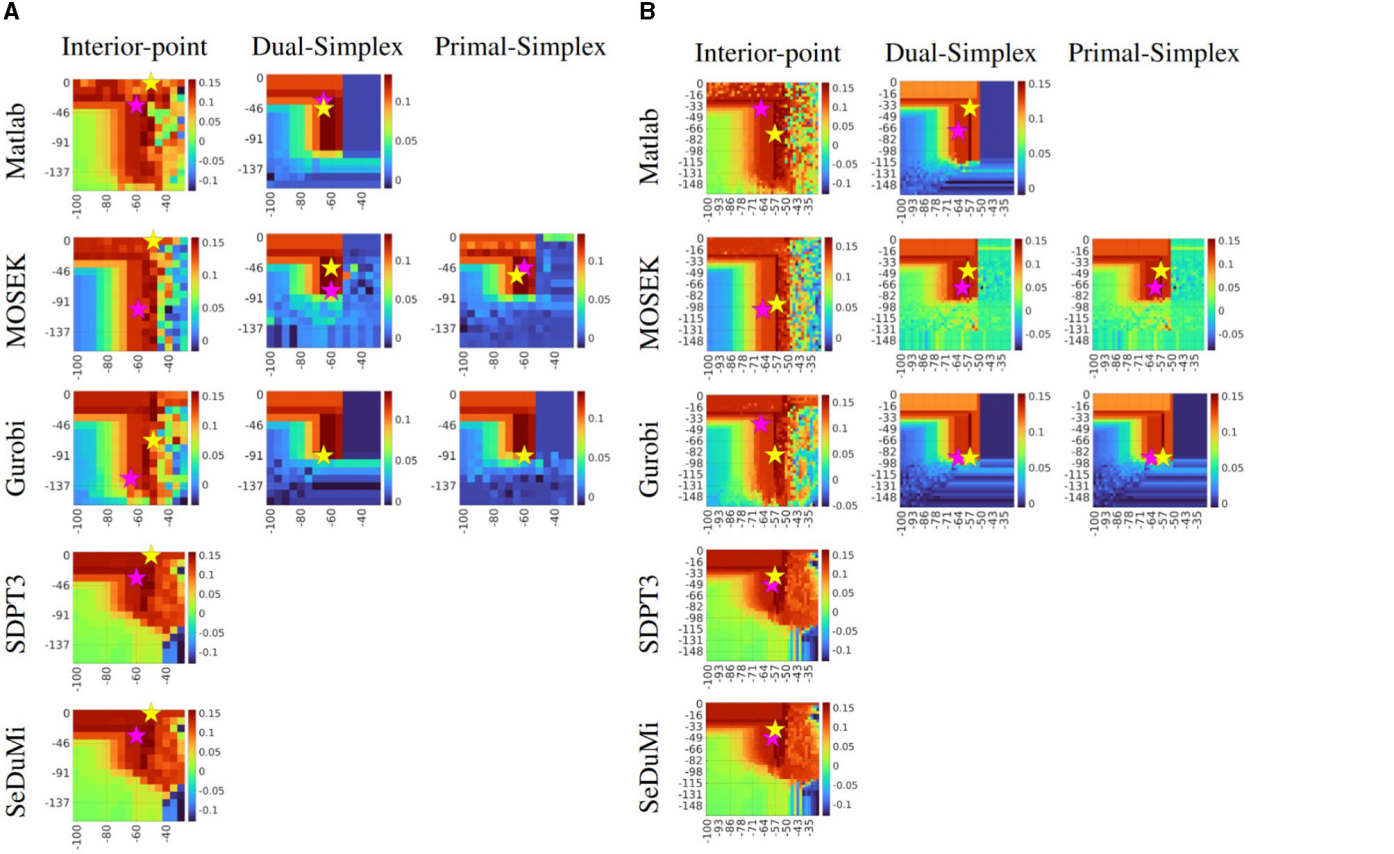
Performance comparison of the exhaustive search for solving the L1-norm fitted and penalized (L1L1) optimization method with current density Γ as the objective function. The coarse **(A)** search space *κ* = 15 produces 225 evaluations, and the finer **(B)** search space *κ* = 40 with 1600 evaluations, both using regularization parameter *α* (x-axis) and the nuisance threshold level *ε* (y-axis). The candidate solutions with respect to current density Γ_max_ and focality Θ_max_ are marked with a yellow and purple star, respectively. The maximizers are generally found around from area in which α is between −71 to −50 dB dB (decibels) and *ε* from 0 to −98 dB. Notice that in the case of using the Gurobi package, with a Simplex algorithm, both optimal candidate solutions for either a stimulus focality or a current density both optimal solutions are taking the same (*α*, *ε*) tuning parameters due to the sharp steepness from the coarse search space. The volumetric current density on every chart is given in Amperes per square meter (A/m^2^).

The exhaustive search proved to be a reliable method for experimental benchmarking when the required tES montage requires careful design for clinical applications. By presenting the exhaustive search results in the form of a heatmap with a coarse grid of *κ* = 15 (Figure 3A), we can pinpoint the (α, ε) region where the focused current amplitude reaches its maximum. Despite a significantly increased number of evaluations, with a finer grid of *κ* = 40 (Figure 3B), we can further determine a more detailed optimal area. This area corresponds to the Cartesian product of α_*m*_ ranging from −71 to −50 dB and ε_*n*_ from 0 to −98 dB. In this context, a high current injection montage, denoted by Γ_max_ (yellow star), is positioned at the peak of the amplitude, while focality-based montages, Θ_max_ (purple star), adhere closely. However, these focality-based montages are slightly deviated due to the influence of the nuisance field, despite being relatively close, as determined by a threshold condition corresponding to 75% of the maximum amplitude achievable with the two-patch bipolar tES montage. In comparison between these grid resolutions, one can observe slight enhancements in amplitude, increased optimization accuracy, and improved numerical stability in the latter case. These aspects are far more noticeable with Dual- and Primal-Simplex methods than with the Interior-Point, which yields overall smoother results with fewer drastic deviations.

**Figure 4:**
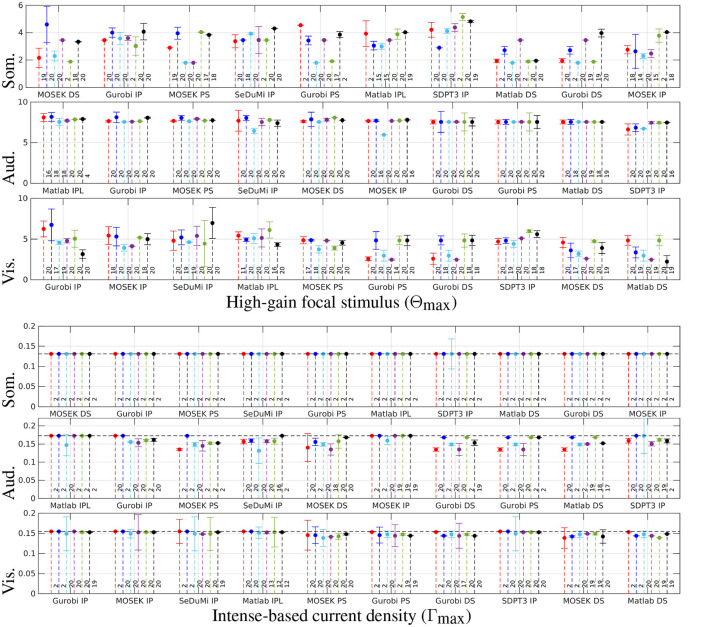
Stem plots results from the two-stage metaheuristic lattice search using exhaustive search with coarse grid of *κ* = 15 (red) and fine grid of *κ* = 40 (blue), and a recursive one with $\tilde{K}=\{3,5,7,9\}$ (cyan, magenta, green, and black, respectively) considering the somatosensory (Som.), auditory (Aud.) and visual (Vis.) regions of interest. The whiskers in the stem plot indicate a second-order Taylor's polynomial estimate for the maximum deviation within a half-lattice unit distance from the optimizer. The intense current injection (Γ_max_) calculated using the reciprocity principle is shown with a horizontal black dashed line as a reference. The number of non-zero (NNZ) channels in the Transcranial Electrical Stimulation (tES) montage is shown on the right side next to the corresponding stem. The solvers are sorted in descending order from left to right based on their performance with *κ* = 40.

Figure 4 delineates the performance nuances among optimization strategies. The whiskers along the stems signify a second-order Taylor's polynomial estimate, reflecting the maximum deviation within half lattice units distance from the optimizer. The reciprocity principle reference for Γ_max_ is represented by a horizontal black dashed line, and the number of non-zero (NNZ) channels required for a tES montage is depicted on the right side of each corresponding stem. The solvers are sorted in ascending order based on their performance, with the exhaustive search *κ* = 40 grid (blue) serving as the point of reference. Both the direct and recursive search techniques adeptly uncover optimal (α, ε) solutions for Θ_max_ and Γ_max_, yielding a substantial reduction in total computing time compared to the specified hyperparameter space.

Due to the heuristic nature of the direct search, and to assert the efficacy of the said technique, we performed a series of trial runs by setting the initial point to the center of the search space. In these trials, the number of objective function evaluations varied, ranging from 25 to 54 trials, with a 33.8 mean among the evaluations (see Table 2). While the number of function evaluations was slightly higher, the quality of the results was nearly on par with those obtained with the recursive search with a search window of $\tilde{K}=3$. With IP solvers, the search runs were mostly successful, while PS and DS tended to fail to find a feasible optimizer candidate.

**Table 2:** Comparison of stimulation focality Θ_max_ and intensity Γ_max_ results obtained between exhaustive, direct, and recursive search methods.

					**Somatosensory**		**Auditory**		**Visual**	
**Search**	**Resolution**	**Levels**	**Window**	**Evaluations**	Θ_max_	Γ_max_	Θ_max_	Γ_max_	Θ_max_	Γ_max_
Exhaustive	Fixed	-	15	225	2.93	0.1315	6.47	0.1486	4.98	0.1488
Exhaustive	Fixed	-	40	1600	3.93	0.1315	7.69	0.1725	4.98	0.1574
Direct	Adaptive	-	-	28-54*	3.45	0.1315	7.75	0.1725	4.99	0.1488
Recursive	Adaptive	3	3	27	2.99	0.1315	6.47	0.1486	5.11	0.1514
	Adaptive	3	5	75	3.45	0.1315	6.92	0.1725	5.17	0.1545
	Adaptive	3	7	147	3.88	0.1315	7.10	0.1725	5.17	0.1545
	Adaptive	3	9	243	4.02	0.1315	7.68	0.1725	3.91	0.1545

The behavior of the search space (Resolution), number of resolution levels (Levels), search window size (Window), and the number of objective function evaluations (Evaluation) per optimization run are given. Non-applicable features are marked with the (-) symbol.

^*^ In direct search, the number of objective function evaluations varied between different optimization runs.

**Figure 5:**
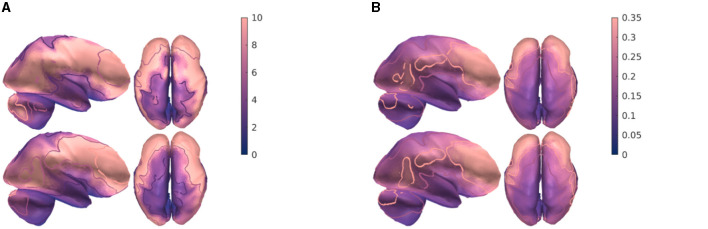
Comparison of the topographical maps for **(A)** maximum focality Θ_max_ and **(B)** current density Γ_max_ using exhaustive search *κ* = 15 (top-row), and recursion with $\tilde{K}=3$ (bottom-row). The contours show 20, 40, and 75% equicurves with respect to their maximum entry. Maps have been computed using the MOSEK with the Interior-point method.

Due to its relatively fast performance among interior-point methods, we applied MOSEK IP to evaluate topographical maps of stimulus focality Θ_max_ (Figure 5A) and current density Γ_max_ (Figure 5B) for an exhaustive search *κ* = 15 and a recursive search $\tilde{K}=3$. Overall, the results of the recursion were close to the outcome of the exhaustive search. Thus, the topographical differences between the different approaches of this study were observed to be minor.

**Figure 6:**
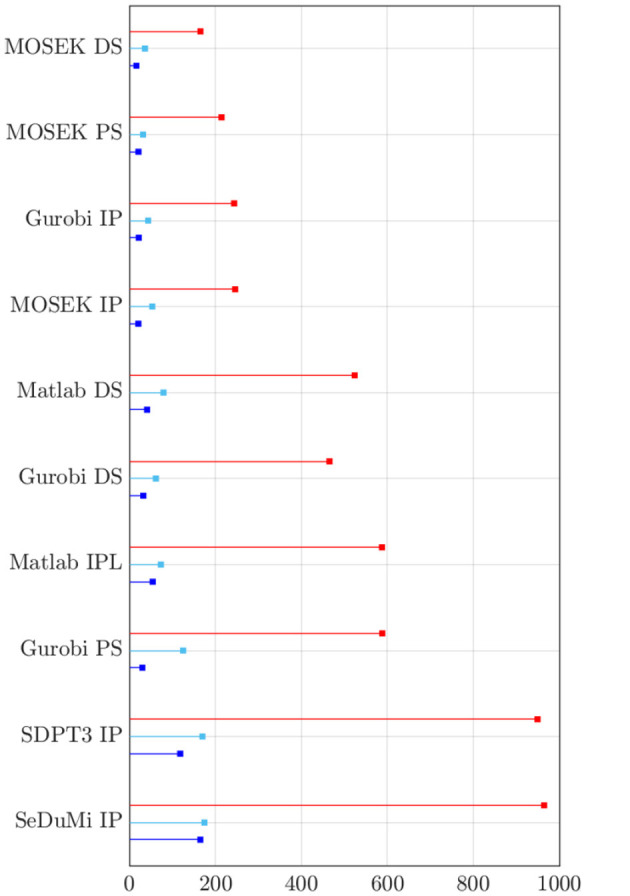
Total amount of computing time for finding the most fitting candidate solution through the two-stage metaheuristic lattice search on every optimization solver and method in this study. In order of stem (top-to-bottom): exhaustive search with search space *κ* = 15 (red), direct search (cyan), and recursive search with $\tilde{K}=3$ (blue). Noticeably, the non-commercial solvers from CVX (SDPT3 and SeDuMi) are significantly slower than those produced by Matlab, Gurobi and MOSEK.

By limiting the search space only to a narrower subspace is a simple countermeasure for dealing with the disadvantages of the exhaustive search. With a *κ* = 15 grid as a reference, it can take approximately 850 seconds to perform a complete search for the first stage, while the second stage only takes roughly 15% of that time since it uses a limited lead field following from the limited number of active electrodes. As an alternative approach, the direct and recursive search seemed to perform well compared to the number of objective function evaluations made during the search process (Figure 6). In particular, MOSEK turned out to be the superior choice, with MOSEK DS being the fastest one. The computing time for Gurobi IP was close to that of MOSEK IP, and Gurobi DS, PS, and Matlab IPL and DS required approximately three times the time. The slowest-performing SDPT3 and SeDuMi took as much as six times the run time of MOSEK IP. Overall, the simplex methods applied to the L1L1 optimization scheme deliver faster yet less accurate solutions than Interior-Point (IP) for focality-based montages, while minor differences can be found for intensity-based solutions.

## 4 Discussion

In this study, we analyzed the numerical and computational performance of exhaustive search, direct search, and recursive search techniques to find an optimal stimulation focality Θ and current density Γ for solving the L1L1 optimization problem for non-invasive transcranial electrical stimulation (tES) current injection. This analysis was motivated by our earlier results in (Galaz Prieto et al., 2022) which suggested that the L1L1 method provides a theoretically attractive approach for obtaining a high-gain focal stimulus as compared to complex L2-norm fitting and regularized least squares techniques (Dmochowski et al., 2011; Wagner et al., 2016).

The reciprocity principle, as outlined by Fernandez-Corazza et al. (2020), served as a reference technique. Its validity was shown for the present tES lead field matrix **L** (see Section 2.3.1). When focusing on a specific target region, the current injection pattern from a two-patch tES montage aligns with the maximum intensity achievable through this principle. Essentially, this involves selecting the two electrodes with the highest absolute back-projected currents. With the absence of nuisance field constraints, the L1L1 solution was observed to agree with the reciprocity principle if the aforementioned algorithmic aspects were handled appropriately.

Decisive aspects for a successful outcome of L1L1 were found to be the choice of the optimization package, algorithm, and search routine, which significantly affect both the performance of the metaheuristic optimization process and output. To enlighten this aspect, we covered the performance of several Interior-Point (IP) (Mehrotra, 1992), Dual-Simplex (DS), and Primal-Simplex (PS) (Boyd and Vandenberghe, 2004) methods from different open-source and commercial optimization toolboxes. We tested the L1L1 method using the commercial solvers of MOSEK Optimization Suite (Release 9) (Mosek, 2019) and Gurobi Optimization (9.5.1) (Gurobi Optimization LLC, 2022), and compared them to the open-source alternatives (Grant and Boyd, 2014) SDPT3 (4.0) (Tütüncü et al., 2003) and SeDuMi (1.3.5) (Sturm, 1999; Frenk et al., 2000; Polik et al., 2007) as well as Matlab R2020b's (MathWorks) Interior-Point-Legacy (IPL) algorithm, which originates from the open LIPSOL (Zhang, 1999) toolbox. We selected the IPL algorithm since we experienced stagnation with Matlab's main IP algorithm, which did not return any appropriate results.

Based on the results, we consider Gurobi IP to be the preferable choice in both optimization stages, considering Θ_max_ and Γ_max_ in each tested target region of interest and, as it was also overall the fastest of the IP solvers. While the best-performing solvers show that the L1L1 method is suitable for maximizing focality and intensity, a few did not find the bipolar current pattern that maximizes Γ_max_. Notably, SDTP3 did not find a bipolar pattern at all, verifying our earlier hypothesis (Galaz Prieto et al., 2022) that the performance of L1L1 might be highly solver-based. Part of the discrepancies between the optimization methods can be explained by a different sensitivity with respect to parameter variation or the resolution of the lattice.

From a computational complexity standpoint, the exhaustive search method can be applied for benchmarking purposes. In contrast, a recursive search proves an advantageous alternative and is competitively on par with the direct search technique, each one applied in this study. This equivalence arises from both methods converging toward the most suitable regularization parameter α and nuisance threshold ε values in a comparably controlled manner. Notably, the computational complexity of recursive search remains consistent across various optimization runs, in contrast to the variability observed in the direct search. Results comparable to those obtained through exhaustive search can be attained with a reduced-resolution search window of, say, size $\tilde{K}=3$, representing a substantial acceleration in comparison to exhaustive search. Furthermore, the recursive approach demonstrates both numerical stability and convergence towards exhaustive search results, both at individual data points and in the overall topographical context, as the probing lattice size increases.

### 4.1 Limitations and future work

Unlike earlier linear programming (LP) formulations for tES optimization problems, our use of the metaheuristic process enabled us to explore parameters freely, without imposing rigid *a priori* constraints on the nuisance field, as observed in Wagner et al. (2016). In L1L1, we optimally set the nuisance field through hyperparameter optimization embedded in a two-stage metaheuristic lattice search procedure. Interpreted as an enhancement for localizing both pattern and volumetric density of the stimulus, L1-norm fitting and regularization outperform the least-squares methodology introduced in Dmochowski et al. (2011, 2017). However, this improvement comes at a greater computational cost, prompting our in-depth investigation into various algorithmic aspects of metaheuristic optimization in this study. Our present findings underscore the critical role of computational considerations when integrating hyperparameters and metacriteria into the tES optimization problem, aspects overlooked in the studies mentioned earlier.

Our results concerning L1L1 are limited to numerically simulated tES only, meaning that neither the performance of the method in other modalities than tES nor the effects of uncertainty causing inter-subject variability (Laakso et al., 2015) have not been fully covered yet. Those might include, for example, any discrepancies between the estimated and actual values of electrical conductivity, such as skull conductivity (Schmidt et al., 2015), strategy to specify a montage (Kaufmann et al., 2021), as well as uncertainty about the targeted region in the brain, e.g., a possible spread of an epileptic focus (Simula et al., 2022). While the expected level of uncertainty can be controlled via the range of the hyperparameter ε, a future study on its effect will obviously need to be conducted.

Of the applied liner programming methods, interior-point is an overall preferable option over the simplex methods, which can be considered beneficial characteristic when hardware performance is limited, e.g., for a potential Field-Programmable Gate Array (FPGA) implementation (Bayliss et al., 2006; Gensheimer et al., 2014). Another comparative method, the Alternating Direction Method of Multipliers (ADMM) (Lin et al., 2021), was not included in this investigation as achieving an appropriate convergence seemed more difficult due to its dependence on a step-length parameter. While the current results enlighten how the different algorithms would perform with different nuisance threshold levels, an independent study would be needed to determine the optimal level given the mathematical uncertainty.

Possible future work directions can be to open up the function of L1L1 on a broader scale, this include applying it for deep brain stimulation (DBS), where the electrical stimulus is not transcranial. Likewise, an advanced optimization technique is needed to target subcortical nuclei of the brain; for instance, in the recent study (Anderson et al., 2018), where the Interior-Point algorithm has been applied. Yet another interesting direction is to consider *a priori* information for the design and application of the L1L1 algorithm, for example, an epileptic focus based on non-invasive measurements such as video-EEG of epileptic activity applied to determine approximate stimulation locations. Finally, the mathematical implications of this study can be further enriched by incorporating transcranial direct current stimulation and functional magnetic resonance imaging (tDCS-fMRI) (Esmaeilpour et al., 2020). By utilizing tDCS-fMRI data sets to explore real-time neural changes caused by electrical stimulation–such as in the studies by Callan et al. (2016) for investigating resting state networks linked to visual stimuli, or in the research conducted by Mark et al. (2023) for monitoring brain activity of pilots undergoing aviation training–further enriches the necessity of an effective inverse problem study equipped with optimization methods for simulating and understanding the signal-to-noise (SNR) impacts with a level of mathematical uncertainty, as some of these deficiencies were mentioned on their study limitations.

## Data availability statement

The original contributions presented in the study are included in the article/supplementary material, further inquiries can be directed to the corresponding author.

## Author contributions

FG: Conceptualization, Data curation, Formal analysis, Investigation, Methodology, Software, Visualization, Writing—original draft. MS: Formal analysis, Project administration, Supervision, Validation, Software, Visualization, Writing—original draft, Writing—review & editing. SP: Conceptualization, Data curation, Formal analysis, Funding acquisition, Investigation, Methodology, Project administration, Resources, Software, Supervision, Validation, Visualization, Writing—review & editing.

## Footnotes

^1^ https://brain-development.org/ixi-dataset/

^2^ https://surfer.nmr.mgh.harvard.edu/

^3^ https://github.com/sampsapursiainen/zeffiro_interface
